# (*E*)-*N*′-(4-Nitro­benzyl­idene)-2-(8-quinol­yloxy)acetohydrazide methanol solvate

**DOI:** 10.1107/S1600536809015992

**Published:** 2009-05-07

**Authors:** Chun-Yan Ren

**Affiliations:** a College of Chemistry and Pharmacy, Qingdao Agricultural University, Shandong 266109, People’s Republic of China

## Abstract

In the title compound, C_18_H_14_N_4_O_4_·CH_3_OH, the mean planes of the benzene ring and the quinoline ring system make a dihedral angle of 15.5 (2)°. The methanol solvent mol­ecule forms an O—H⋯N hydrogen bond to the quinoline ring system and accepts an N—H⋯O hydrogen bond from the hydrazide NH group. The mol­ecules lie in layers approximately parallel to (101) and C—H⋯O inter­actions exist between mol­ecules within the layers.

## Related literature

For the coordination chemistry of 8-hydroxy­quinoline and its derivatives, see: Chen & Shi (1998 ▶); Mona & Wageih (2002 ▶). For a related structure, see: Tan (2009 ▶).
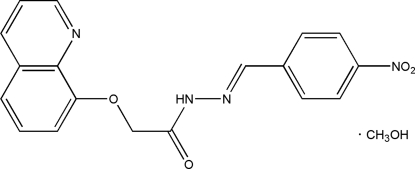


## Experimental

### 

#### Crystal data

C_18_H_14_N_4_O_4_·CH_4_O
*M* *_r_* = 382.37
Monoclinic, 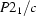

*a* = 11.345 (10) Å
*b* = 11.559 (11) Å
*c* = 16.234 (12) Å
β = 120.06 (5)°
*V* = 1843 (3) Å^3^
*Z* = 4
Mo *K*α radiation
μ = 0.10 mm^−1^
*T* = 295 K
0.20 × 0.18 × 0.16 mm

#### Data collection

Bruker SMART CCD diffractometer
Absorption correction: multi-scan (*SADABS*; Sheldrick, 1996 ▶) *T*_min_ = 0.980, *T*_max_ = 0.984
9196 measured reflections
3258 independent reflections
1872 reflections with *I* > 2σ(*I*)
*R*_int_ = 0.050

#### Refinement

*R*[*F* ^2^ > 2σ(*F* ^2^)] = 0.056
*wR*(*F* ^2^) = 0.195
*S* = 1.00
3258 reflections
255 parameters
H-atom parameters constrained
Δρ_max_ = 0.21 e Å^−3^
Δρ_min_ = −0.26 e Å^−3^

### 

Data collection: *SMART* (Siemens, 1996 ▶); cell refinement: *SAINT* (Siemens, 1996 ▶); data reduction: *SAINT*; program(s) used to solve structure: *SHELXS97* (Sheldrick, 2008 ▶); program(s) used to refine structure: *SHELXL97* (Sheldrick, 2008 ▶); molecular graphics: *SHELXTL* (Sheldrick, 2008 ▶); software used to prepare material for publication: *SHELXTL*.

## Supplementary Material

Crystal structure: contains datablocks global, I. DOI: 10.1107/S1600536809015992/bi2363sup1.cif

Structure factors: contains datablocks I. DOI: 10.1107/S1600536809015992/bi2363Isup2.hkl

Additional supplementary materials:  crystallographic information; 3D view; checkCIF report

## Figures and Tables

**Table 1:** Hydrogen-bond geometry (Å, °)

*D*—H⋯*A*	*D*—H	H⋯*A*	*D*⋯*A*	*D*—H⋯*A*
O5—H5⋯N1	0.82	2.02	2.817 (4)	164
N2—H2⋯O5	0.86	2.08	2.919 (4)	164
C17—H17⋯O3^i^	0.93	2.53	3.287 (5)	139
C18—H18⋯O4^i^	0.93	2.48	3.329 (5)	152
C3—H3⋯O2^ii^	0.93	2.58	3.233 (5)	128

Symmetry codes: (i) 
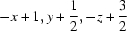
; (ii) 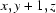.

**Fig. 1.**
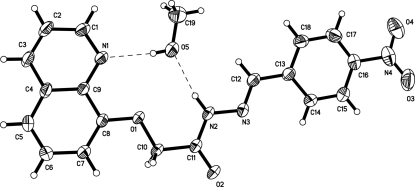
The molecular structure with displacement ellipsoids drawn at the 30% probability level for non-H atoms. The dashed lines indicate hydrogen bonds.

**Fig. 2.**
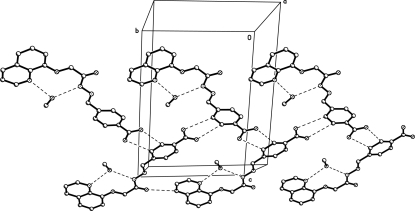
Intermolecular C—H···O interactions (dashed lines). H atoms have been omitted for clarity.

## supplementary crystallographic information

### Comment

8-Hydroxyquinoline and its derivatives are well-known ligands in coordination chemistry (Chen & Shi, 1998; Mona & Wageih, 2002). In our search for new extractants of metal ions and biologically active materials, the title compound has been synthesized. In the crystal structure, all bond lengths and angles are normal and comparable to those in the related compound (*E*)-*N*'-[1-(4-hydroxyphenyl) ethylidene]-2-(quinolin-8-yloxy)acetohydrazide methanol solvate (Tan, 2009). The mean planes of the benzene ring and the quinoline rings make a dihedral angle of 15.5 (2)°. The methanol molecule is linked to the C_18_H_14_N_4_O_4_ molecule *via* intermolecular O—H···N and N—H···O hydrogen bonds (Fig. 1). The molecules lie in layers approximately parallel to (101) and C—H···O interactions exist between molecules.

### Experimental

2-(Quinolin-8-yloxy)acetohydrazide (2.18 g, 10 mmol), 4-nitrobenzaldehyde (1.51 g, 10 mmol), ethanol (40 ml) and some drops of acetic acid were added to a 100 ml flask, and refluxed for 3 h. After cooling to room temperature, the mixture was filtered. Colourless single crystals suitable for X-ray diffraction were obtained by slow evaporation of an acetone-methanol (1:2, *v*/*v*) solution over a period of 3 d.

### Refinement

All H atoms were visible in a difference Fourier map, but were placed in calculated positions with C—H = 0.93 Å for aryl, 0.97 Å for methylene and 0.96 Å for the methyl H atoms, O—H = 0.82 Å and N—H = 0.86 Å, and refined as riding with *U*_iso_(H) = 1.2*U*_eq_(C/N), or 1.5*U*_eq_(C) for the methyl groups, and 1.5*U*_eq_(O).

### Crystal data

**Table:**

C_18_H_14_N_4_O_4_·CH_4_O	*F*(000) = 800
*M**_r_* = 382.37	*D*_x_ = 1.378 Mg m^−^^3^
Monoclinic, *P*2_1_/*c*	Mo *K*α radiation, λ = 0.71073 Å
Hall symbol: -P 2ybc	Cell parameters from 1871 reflections
*a* = 11.345 (10) Å	θ = 2.3–22.8°
*b* = 11.559 (11) Å	µ = 0.10 mm^−^^1^
*c* = 16.234 (12) Å	*T* = 295 K
β = 120.06 (5)°	Block, colorless
*V* = 1843 (3) Å^3^	0.20 × 0.18 × 0.16 mm
*Z* = 4	

### Data collection

**Table:**

Bruker SMART CCD diffractometer	3258 independent reflections
Radiation source: fine-focus sealed tube	1872 reflections with *I* > 2σ(*I*)
graphite	*R*_int_ = 0.050
φ and ω scans	θ_max_ = 25.1°, θ_min_ = 2.1°
Absorption correction: multi-scan (*SADABS*; Sheldrick, 1996)	*h* = −13→10
*T*_min_ = 0.980, *T*_max_ = 0.984	*k* = −12→13
9196 measured reflections	*l* = −17→19

### Refinement

**Table:**

Refinement on *F*^2^	Primary atom site location: structure-invariant direct methods
Least-squares matrix: full	Secondary atom site location: difference Fourier map
*R*[*F*^2^ > 2σ(*F*^2^)] = 0.056	Hydrogen site location: inferred from neighbouring sites
*wR*(*F*^2^) = 0.195	H-atom parameters constrained
*S* = 1.00	*w* = 1/[σ^2^(*F*_o_^2^) + (0.1106*P*)^2^ + 0.0143*P*] where *P* = (*F*_o_^2^ + 2*F*_c_^2^)/3
3258 reflections	(Δ/σ)_max_ < 0.001
255 parameters	Δρ_max_ = 0.21 e Å^−^^3^
0 restraints	Δρ_min_ = −0.26 e Å^−^^3^

### Special details

**Table:**

Geometry. All e.s.d.'s (except the e.s.d. in the dihedral angle between two l.s. planes) are estimated using the full covariance matrix. The cell e.s.d.'s are taken into account individually in the estimation of e.s.d.'s in distances, angles and torsion angles; correlations between e.s.d.'s in cell parameters are only used when they are defined by crystal symmetry. An approximate (isotropic) treatment of cell e.s.d.'s is used for estimating e.s.d.'s involving l.s. planes.
Refinement. Refinement of *F*^2^ against ALL reflections. The weighted *R*-factor *wR* and goodness of fit *S* are based on *F*^2^, conventional *R*-factors *R* are based on *F*, with *F* set to zero for negative *F*^2^. The threshold expression of *F*^2^ > σ(*F*^2^) is used only for calculating *R*-factors(gt) *etc*. and is not relevant to the choice of reflections for refinement. *R*-factors based on *F*^2^ are statistically about twice as large as those based on *F*, and *R*- factors based on ALL data will be even larger.

### Fractional atomic coordinates and isotropic or equivalent isotropic displacement parameters (Å^2^)

**Table:**

	*x*	*y*	*z*	*U*_iso_*/*U*_eq_	
O1	0.1624 (2)	0.23691 (14)	1.11105 (14)	0.0547 (6)	
O2	0.1325 (2)	−0.07065 (16)	1.10099 (16)	0.0694 (7)	
O3	0.4318 (3)	−0.4685 (3)	0.7787 (2)	0.1065 (10)	
O4	0.5020 (4)	−0.3368 (3)	0.7175 (2)	0.1177 (12)	
O5	0.2556 (3)	0.27429 (19)	0.9641 (2)	0.0938 (9)	
H5	0.2540	0.3180	1.0033	0.141*	
N1	0.2147 (3)	0.45024 (19)	1.06723 (17)	0.0541 (7)	
N2	0.2130 (3)	0.05140 (19)	1.02969 (17)	0.0547 (7)	
H2	0.2310	0.1214	1.0219	0.066*	
N3	0.2403 (3)	−0.0398 (2)	0.98661 (17)	0.0538 (7)	
N4	0.4508 (3)	−0.3671 (3)	0.7651 (2)	0.0834 (9)	
C1	0.2366 (3)	0.5556 (3)	1.0457 (2)	0.0621 (9)	
H1	0.2848	0.5621	1.0135	0.075*	
C2	0.1921 (4)	0.6582 (2)	1.0679 (2)	0.0652 (9)	
H2A	0.2101	0.7298	1.0504	0.078*	
C3	0.1227 (4)	0.6509 (2)	1.1152 (2)	0.0602 (9)	
H3	0.0909	0.7176	1.1296	0.072*	
C4	0.0985 (3)	0.5417 (2)	1.14255 (19)	0.0510 (8)	
C5	0.0322 (3)	0.5278 (3)	1.1966 (2)	0.0605 (9)	
H5A	−0.0014	0.5924	1.2123	0.073*	
C6	0.0173 (3)	0.4215 (3)	1.2255 (2)	0.0618 (9)	
H6	−0.0221	0.4141	1.2635	0.074*	
C7	0.0611 (3)	0.3223 (2)	1.1981 (2)	0.0569 (8)	
H7	0.0483	0.2498	1.2172	0.068*	
C8	0.1224 (3)	0.3306 (2)	1.14367 (19)	0.0474 (7)	
C9	0.1459 (3)	0.4422 (2)	1.11638 (18)	0.0456 (7)	
C10	0.1252 (3)	0.1263 (2)	1.1307 (2)	0.0564 (8)	
H10A	0.0283	0.1263	1.1084	0.068*	
H10B	0.1728	0.1145	1.1990	0.068*	
C11	0.1570 (3)	0.0270 (2)	1.0849 (2)	0.0521 (8)	
C12	0.2988 (3)	−0.0149 (2)	0.9392 (2)	0.0543 (8)	
H12	0.3186	0.0619	0.9339	0.065*	
C13	0.3350 (3)	−0.1051 (2)	0.89341 (19)	0.0511 (7)	
C14	0.2917 (3)	−0.2196 (2)	0.8882 (2)	0.0602 (8)	
H14	0.2367	−0.2386	0.9135	0.072*	
C15	0.3289 (3)	−0.3049 (3)	0.8461 (2)	0.0630 (9)	
H15	0.2996	−0.3808	0.8429	0.076*	
C16	0.4108 (3)	−0.2748 (3)	0.8090 (2)	0.0604 (8)	
C17	0.4521 (3)	−0.1636 (3)	0.8101 (2)	0.0663 (9)	
H17	0.5048	−0.1452	0.7828	0.080*	
C18	0.4146 (3)	−0.0785 (3)	0.8526 (2)	0.0610 (9)	
H18	0.4427	−0.0026	0.8539	0.073*	
C19	0.3321 (4)	0.3241 (3)	0.9278 (3)	0.0858 (12)	
H19A	0.2805	0.3851	0.8845	0.129*	
H19B	0.3535	0.2664	0.8948	0.129*	
H19C	0.4149	0.3552	0.9791	0.129*	

### Atomic displacement parameters (Å^2^)

**Table:**

	*U*^11^	*U*^22^	*U*^33^	*U*^12^	*U*^13^	*U*^23^
O1	0.0850 (16)	0.0254 (10)	0.0755 (13)	−0.0023 (9)	0.0564 (13)	−0.0003 (9)
O2	0.1058 (19)	0.0270 (11)	0.1018 (17)	−0.0034 (10)	0.0717 (16)	0.0019 (10)
O3	0.121 (3)	0.069 (2)	0.136 (3)	0.0176 (17)	0.069 (2)	−0.0199 (19)
O4	0.142 (3)	0.125 (3)	0.131 (3)	0.015 (2)	0.102 (3)	−0.0246 (19)
O5	0.158 (3)	0.0435 (14)	0.135 (2)	0.0016 (14)	0.115 (2)	0.0028 (14)
N1	0.0780 (19)	0.0345 (13)	0.0612 (15)	−0.0063 (11)	0.0433 (15)	0.0001 (11)
N2	0.0777 (19)	0.0262 (12)	0.0716 (16)	−0.0015 (11)	0.0458 (15)	−0.0002 (11)
N3	0.0709 (18)	0.0345 (13)	0.0654 (15)	0.0027 (11)	0.0412 (15)	−0.0025 (11)
N4	0.080 (2)	0.092 (3)	0.082 (2)	0.0172 (19)	0.0434 (19)	−0.016 (2)
C1	0.089 (3)	0.0418 (18)	0.070 (2)	−0.0116 (16)	0.050 (2)	0.0019 (15)
C2	0.098 (3)	0.0310 (17)	0.0654 (19)	−0.0110 (15)	0.040 (2)	0.0003 (14)
C3	0.087 (3)	0.0307 (16)	0.0627 (19)	−0.0032 (14)	0.037 (2)	−0.0045 (14)
C4	0.068 (2)	0.0328 (16)	0.0490 (16)	−0.0031 (13)	0.0269 (16)	−0.0067 (12)
C5	0.077 (2)	0.0457 (19)	0.069 (2)	0.0000 (15)	0.0442 (19)	−0.0144 (15)
C6	0.082 (3)	0.0512 (19)	0.072 (2)	−0.0057 (16)	0.053 (2)	−0.0076 (16)
C7	0.083 (2)	0.0366 (16)	0.0660 (19)	−0.0038 (14)	0.0488 (19)	0.0007 (14)
C8	0.065 (2)	0.0309 (15)	0.0525 (16)	−0.0004 (12)	0.0337 (16)	−0.0022 (12)
C9	0.062 (2)	0.0324 (15)	0.0469 (15)	−0.0053 (12)	0.0306 (15)	−0.0034 (12)
C10	0.088 (2)	0.0266 (14)	0.0736 (19)	−0.0052 (14)	0.0549 (19)	0.0000 (13)
C11	0.069 (2)	0.0314 (16)	0.0642 (19)	−0.0019 (13)	0.0396 (18)	−0.0020 (13)
C12	0.069 (2)	0.0382 (17)	0.0608 (18)	−0.0017 (14)	0.0365 (18)	0.0021 (13)
C13	0.060 (2)	0.0408 (17)	0.0543 (17)	−0.0012 (13)	0.0301 (16)	0.0014 (13)
C14	0.077 (2)	0.0435 (17)	0.080 (2)	−0.0036 (15)	0.054 (2)	−0.0005 (16)
C15	0.074 (2)	0.0431 (18)	0.079 (2)	−0.0003 (15)	0.044 (2)	−0.0031 (16)
C16	0.062 (2)	0.059 (2)	0.0636 (19)	0.0077 (16)	0.0339 (18)	−0.0069 (16)
C17	0.071 (2)	0.074 (2)	0.069 (2)	−0.0113 (17)	0.046 (2)	−0.0087 (18)
C18	0.069 (2)	0.0535 (19)	0.070 (2)	−0.0128 (15)	0.0422 (19)	−0.0046 (16)
C19	0.100 (3)	0.083 (3)	0.086 (3)	−0.002 (2)	0.055 (3)	0.005 (2)

### Geometric parameters (Å, °)

**Table:**

O1—C8	1.378 (3)	C6—C7	1.407 (4)
O1—C10	1.431 (3)	C6—H6	0.930
O2—C11	1.222 (3)	C7—C8	1.375 (4)
O3—N4	1.232 (4)	C7—H7	0.930
O4—N4	1.227 (4)	C8—C9	1.431 (4)
O5—C19	1.394 (4)	C10—C11	1.506 (4)
O5—H5	0.820	C10—H10A	0.970
N1—C1	1.324 (4)	C10—H10B	0.970
N1—C9	1.371 (4)	C12—C13	1.455 (4)
N2—C11	1.362 (4)	C12—H12	0.930
N2—N3	1.383 (3)	C13—C18	1.395 (4)
N2—H2	0.860	C13—C14	1.399 (4)
N3—C12	1.275 (4)	C14—C15	1.380 (4)
N4—C16	1.476 (4)	C14—H14	0.930
C1—C2	1.406 (4)	C15—C16	1.381 (4)
C1—H1	0.930	C15—H15	0.930
C2—C3	1.350 (5)	C16—C17	1.366 (4)
C2—H2A	0.930	C17—C18	1.385 (4)
C3—C4	1.411 (4)	C17—H17	0.930
C3—H3	0.930	C18—H18	0.930
C4—C9	1.421 (4)	C19—H19A	0.960
C4—C5	1.422 (4)	C19—H19B	0.960
C5—C6	1.355 (4)	C19—H19C	0.960
C5—H5A	0.930		
			
C8—O1—C10	115.2 (2)	O1—C10—C11	113.6 (2)
C19—O5—H5	109.5	O1—C10—H10A	108.8
C1—N1—C9	116.9 (2)	C11—C10—H10A	108.8
C11—N2—N3	118.1 (2)	O1—C10—H10B	108.8
C11—N2—H2	121.0	C11—C10—H10B	108.8
N3—N2—H2	120.9	H10A—C10—H10B	107.7
C12—N3—N2	116.6 (2)	O2—C11—N2	124.3 (3)
O4—N4—O3	124.4 (3)	O2—C11—C10	117.5 (3)
O4—N4—C16	117.1 (4)	N2—C11—C10	118.2 (2)
O3—N4—C16	118.5 (3)	N3—C12—C13	120.8 (3)
N1—C1—C2	124.7 (3)	N3—C12—H12	119.6
N1—C1—H1	117.6	C13—C12—H12	119.6
C2—C1—H1	117.6	C18—C13—C14	118.1 (3)
C3—C2—C1	118.7 (3)	C18—C13—C12	120.0 (3)
C3—C2—H2A	120.7	C14—C13—C12	121.9 (3)
C1—C2—H2A	120.7	C15—C14—C13	121.4 (3)
C2—C3—C4	119.8 (3)	C15—C14—H14	119.3
C2—C3—H3	120.1	C13—C14—H14	119.3
C4—C3—H3	120.1	C14—C15—C16	118.3 (3)
C3—C4—C9	117.9 (3)	C14—C15—H15	120.8
C3—C4—C5	122.8 (3)	C16—C15—H15	120.8
C9—C4—C5	119.3 (3)	C17—C16—C15	122.3 (3)
C6—C5—C4	120.8 (3)	C17—C16—N4	120.0 (3)
C6—C5—H5A	119.6	C15—C16—N4	117.8 (3)
C4—C5—H5A	119.6	C16—C17—C18	119.0 (3)
C5—C6—C7	120.3 (3)	C16—C17—H17	120.5
C5—C6—H6	119.9	C18—C17—H17	120.5
C7—C6—H6	119.9	C17—C18—C13	120.9 (3)
C8—C7—C6	121.3 (3)	C17—C18—H18	119.6
C8—C7—H7	119.4	C13—C18—H18	119.6
C6—C7—H7	119.4	O5—C19—H19A	109.5
C7—C8—O1	124.2 (2)	O5—C19—H19B	109.5
C7—C8—C9	119.6 (2)	H19A—C19—H19B	109.5
O1—C8—C9	116.2 (2)	O5—C19—H19C	109.5
N1—C9—C4	122.0 (2)	H19A—C19—H19C	109.5
N1—C9—C8	119.3 (2)	H19B—C19—H19C	109.5
C4—C9—C8	118.7 (3)		
			
C11—N2—N3—C12	−176.7 (3)	O1—C8—C9—C4	175.8 (3)
C9—N1—C1—C2	−1.3 (5)	C8—O1—C10—C11	173.7 (3)
N1—C1—C2—C3	0.4 (5)	N3—N2—C11—O2	2.0 (5)
C1—C2—C3—C4	1.1 (5)	N3—N2—C11—C10	−179.0 (3)
C2—C3—C4—C9	−1.7 (5)	O1—C10—C11—O2	177.6 (3)
C2—C3—C4—C5	176.8 (3)	O1—C10—C11—N2	−1.4 (4)
C3—C4—C5—C6	−176.6 (3)	N2—N3—C12—C13	178.4 (3)
C9—C4—C5—C6	1.9 (5)	N3—C12—C13—C18	−171.6 (3)
C4—C5—C6—C7	−3.3 (5)	N3—C12—C13—C14	8.8 (5)
C5—C6—C7—C8	1.5 (5)	C18—C13—C14—C15	1.6 (5)
C6—C7—C8—O1	−177.1 (3)	C12—C13—C14—C15	−178.7 (3)
C6—C7—C8—C9	1.8 (5)	C13—C14—C15—C16	0.1 (5)
C10—O1—C8—C7	5.2 (4)	C14—C15—C16—C17	−2.0 (5)
C10—O1—C8—C9	−173.7 (3)	C14—C15—C16—N4	179.4 (3)
C1—N1—C9—C4	0.6 (4)	O4—N4—C16—C17	−11.5 (5)
C1—N1—C9—C8	−178.4 (3)	O3—N4—C16—C17	168.2 (4)
C3—C4—C9—N1	0.9 (4)	O4—N4—C16—C15	167.1 (3)
C5—C4—C9—N1	−177.7 (3)	O3—N4—C16—C15	−13.2 (5)
C3—C4—C9—C8	179.9 (3)	C15—C16—C17—C18	2.1 (5)
C5—C4—C9—C8	1.4 (4)	N4—C16—C17—C18	−179.3 (3)
C7—C8—C9—N1	175.9 (3)	C16—C17—C18—C13	−0.3 (5)
O1—C8—C9—N1	−5.1 (4)	C14—C13—C18—C17	−1.5 (5)
C7—C8—C9—C4	−3.2 (4)	C12—C13—C18—C17	178.8 (3)

### Hydrogen-bond geometry (Å, °)

**Table:**

*D*—H···*A*	*D*—H	H···*A*	*D*···*A*	*D*—H···*A*
O5—H5···N1	0.82	2.02	2.817 (4)	164
N2—H2···O5	0.86	2.08	2.919 (4)	164
C17—H17···O3^i^	0.93	2.53	3.287 (5)	139
C18—H18···O4^i^	0.93	2.48	3.329 (5)	152
C3—H3···O2^ii^	0.93	2.58	3.233 (5)	128

Symmetry codes: (i) −*x*+1, *y*+1/2, −*z*+3/2; (ii) *x*, *y*+1, *z*.
